# Association between Serum Calcium and Risk of Cardiometabolic Disease among Community-dwelling Adults in Taiwan

**DOI:** 10.1038/s41598-020-60209-w

**Published:** 2020-02-21

**Authors:** Cheng-Wai Chou, Wen-Hui Fang, Yuan-Yuei Chen, Chung-Ching Wang, Tung-Wei Kao, Chen-Jung Wu, Wei-Liang Chen

**Affiliations:** 1 Division of Family Medicine, Department of Family and Community Medicine, Tri-Service General Hospital; and School of Medicine, National Defense Medical Center, Taipei, Taiwan, Republic of China; 2 Department of Otorhinolaryngology Head and Neck Surgery, Taipei Veterans General Hospital, Taipei, Taiwan, Republic of China; 3 Division of Geriatric Medicine, Department of Family and Community Medicine, Tri-Service General Hospital; and School of Medicine, National Defense Medical Center, Taipei, Taiwan, Republic of China; 4 Graduate Institute of Clinical Medical, College of Medicine, National Taiwan University, Taipei, Taiwan, Republic of China; 5 Division of Family Medicine, Department of Community Medicine, Taoyuan Armed Forces General Hospital, Taoyuan, Taiwan, Republic of China

**Keywords:** Public health, Risk factors

## Abstract

Serum calcium, although only comprising 1% of total body calcium, is involved in intracellular signal pathways, vascular dilatation/constriction, and muscle contraction, which are crucial for insulin secretion, cholesterol catabolism, and blood pressure regulation. As far as we know, research on the relationship between serum calcium level and metabolic syndrome (MetS), diabetes, and hypertension in one longitudinal study is rare. Owing to the crucial role of serum calcium in human cardiometabolic physiology and lack of related study so far, this study aims to describe the relationship between serum calcium level and the incidence of MetS, diabetes, and hypertension. During the period 2010–2016, there are two parts to our study: cross-sectional analysis and longitudinal analysis. Logistic regression was applied for cross-sectional analysis of the association between serum calcium level or albumin-corrected calcium (ACCA) and the prevalence of MetS, diabetes, or hypertension. Receiver operating characteristic (ROC) curve analysis was used for calculating of optimal cut-off value of serum calcium and ACCA. Cox proportional regression for development of MetS, diabetes, and hypertension according to different cut-off values of serum calcium level and ACCA were conducted. At baseline, there were 27,364 participants in our study. Serum calcium level had positive association with diabetes in the total population, male, and female. ACCA level had positive association with diabetes in the total population, male, and female. In unadjusted and multivariate Cox proportional hazard models, serum calcium level was associated with increased risk of incident MetS in the total population and male. ACCA was associated with increased risk of incident MetS in the total population and male. ACCA was associated with increased risk of incident diabetes in the total population and male participants. This study describes the relationship between serum calcium level and the incidence of MetS, diabetes, and hypertension. Higher serum calcium level is associated with increased risk of MetS, diabetes, and hypertension.

## Introduction

Metabolic syndrome (MetS), diabetes mellitus, and hypertension are three major health challenges in industrialized countries worldwide. According to the estimation of the International Diabetes Federation (IDF), the prevalence of MetS around the world is 25%^1^. The estimated number of people with diabetes in 2030 will be 366 million, and the number of adults with hypertension in 2025 is predicted to be 1.56 billion^2^. Serum calcium, although only comprising 1% of total body calcium, is involved in intracellular signal pathways, vascular dilatation/constriction, and muscle contraction^3^. These functions are crucial for insulin secretion, cholesterol catabolism, and blood pressure regulation, which are important etiologies of MetS, diabetes, and hypertension. Hypertension occurs in 50% to 80% of patients with type 2 diabetes in the United States^4^. This high overlap rate can be explained by the common etiologies and mechanisms between MetS, diabetes, and hypertension, such as insulin resistance, inflammation, oxidative stress, and obesity^5^. Patients often suffer more than one of these diseases, accelerating the onset of complications. Owing to the crucial role serum calcium plays in MetS, diabetes, hypertension and the extensive overlap of these diseases, a longitudinal cohort study to estimate how well serum calcium predicts their incidences wound be interesting.

In Germany, Spain and Italy, for the healthcare system, the economic burden of MetS in patients with hypertension was estimated at €24,427, €1,900 and €4,877 million and are expected to rise by 59%, 179% and 157% respectively by 2020, most of the costs would be used on prevention of type 2 diabetes and cardiovascular incidents^6^. In India, the age-standardized cardiovascular disease death rate is estimated to be 272 per 100,000 population, this number exceeded the global average. In addition, over the past two decades, there is a 59% increase in premature mortality because of cardiovascular disease in India^7^. In 2009, high blood glucose accounted for 14,900 deaths, or 10.4% of all deaths in Taiwan, whereas high blood pressure accounted for 11,190 deaths^8^. More studies should be conducted to provide better understanding of cardiometabolic diseases, since they have posed a very serious threat to the human health worldwide and a heavy burden to the economy. As far as we know, there have been researches on association between serum calcium level and MetS, diabetes, and hypertension respectively, but simultaneous research on the relationship between serum calcium level and MetS, diabetes, and hypertension in one longitudinal study is rare^9–13^. This longitudinal study is a pioneering study that simultaneously considers incident MetS, diabetes, and hypertension according to serum calcium level. Calcium level is corrected using albumin to better present the actual calcium concentration in serum.

## Results

### Age and metabolic parameters as related to serum calcium level

Age and metabolic parameters as related to serum calcium level are shown in Table 1. Subjects with serum calcium or ACCA level greater than the cut-off values had higher body mass index (BMI), total cholesterol, triglycerides, low-density lipid cholesterol (LDL-C), creatinine, uric acid, and albumin.

**Table 1:** Age and metabolic parameters as related to serum calcium level.

**Continuous variables, mean (SD)	Serum calcium >cut-off value (N = 12367)	Serum calcium ≤cut-off value (N = 14997)	P-value
*ACCA (mg/dL)	9.07 (0.29)	8.84 (0.34)	<0.001
Age (years)	42.63 (12.76)	42.65 (13.19)	0.907
*Body mass index (BMI) (kg/m^2^)	24.74 (3.68)	23.25 (4.03)	<0.001
*Total cholesterol (mg/dL)	187.42 (35.79)	184.66 (35.15)	<0.001
*Triglycerides (TG) (mg/dL)	128.79 (96.20)	101.08 (73.89)	<0.001
*Low density lipid cholesterol (LDL-C) (mg/dL)	118.39 (31.82)	111.44 (30.80)	<0.001
*Creatinine (mg/dL)	0.92 (0.32)	0.71 (0.26)	<0.001
*Uric acid (mg/dL)	6.30 (1.34)	4.89 (1.23)	<0.001
*Albumin (g/dL)	4.60 (0.26)	4.40 (0.28)	<0.001
	ACCA > cut-off value (N = 14284)	ACCA ≤ cut-off value (N = 13080)	
*Serum calcium (mg/dL)	9.46 (0.35)	9.19 (0.36)	<0.001
Age (years)	44.13 (13.05)	41.01 (12.75)	0.907
*Body mass index (BMI) (kg/m^2^)	24.87 (3.70)	22.88 (3.95)	<0.001
*Total cholesterol (mg/dL)	186.89 (35.75)	184.79 (35.095)	<0.001
*Triglycerides (TG) (mg/dL)	130.08 (98.58)	95.55 (64.59)	<0.001
*Low density lipid cholesterol (LDL-C) (mg/dL)	117.99 (31.53)	110.60 (30.89)	<0.001
*Creatinine (mg/dL)	0.92 (0.35)	0.68 (0.19)	<0.001
*Uric acid (mg/dL)	6.26 (1.35)	4.73 (1.13)	<0.001
*Albumin (g/dL)	4.53 (0.29)	4.45 (0.28)	<0.001

ACCA, albumin corrected calcium.

*Statistically significant variables es (p value < 0.05) **Student’s t-test.

### Relationship between serum calcium, metabolic syndrome, diabetes, and hypertension

Relationship between serum calcium level and MetS, diabetes, and hypertension were analyzed by logistic regression in Table 2. The statistical significance of the association between serum calcium level and MetS in the total population was at borderline, the odd ratio (OR) was 1.13 (95% CI: 1.00–1.29 P = 0.057) in model 2. Serum calcium level had positive association with diabetes in the total population, male, and female, ORs were 1.40 (95% CI: 1.16–1.68 P < 0.001), 1.35 (95% CI: 1.10–1.67 P = 0.005), and 1.50 (95% CI: 1.03–2.18 P = 0.036) in model 2. Serum calcium level had positive association with hypertension in the total population, OR was 1.14 (95% CI: 1.03–1.27 P = 0.013) in model 2.

**Table 2:** Relationship among serum calcium, MetS, diabetes, and hypertension in the cross-sectional study.

	Metabolic syndrome				Diabetes mellitus				Hypertension			
	Model 1		Model 2		Model 1		Model 2		Model 1		Model 2	
	OR (95% CI)	P value	OR (95% CI)	P value	OR (95% CI)	P value	OR (95% CI)	P value	OR (95% CI)	P value	OR (95% CI)	P value
Total	*1.17 (1.05–1.30)	0.006	1.13 (1.00–1.29)	0.057	*1.32 (1.10–1.57)	0.002	*1.40 (1.16–1.68)	<0.001	*1.37 (1.24–1.52)	<0.001	*1.14 (1.03–1.27)	0.013
Male	1.01 (0.89–1.15)	0.842	1.08 (0.93–1.25)	0.338	*1.23 (1.01–1.50)	0.042	*1.35 (1.10–1.67)	0.005	*1.23 (1.09–1.38)	0.001	1.10 (0.98–1.25)	0.111
Female	*1.52 (1.23–1.90)	<0.001	1.26 (0.96–1.65)	0.095	*1.65 (1.16–2.34)	0.006	*1.50 (1.03–2.18)	0.036	*1.63 (1.33–2.00)	<0.001	1.17 (0.94–1.45)	0.153

MetS, Metabolic syndrome; OR, Odds ratio.

Model 1: Adjusted for age, gender and body mass index (BMI); Model 2: Adjusted for age, gender, BMI, total cholesterol, triglycerides, LDL-C, creatinine, uric acid, serum albumin, proteinuria, and smoking status.

Response variables: MetS or diabetes or hypertension status; Explanatory variables: Serum calcium level (mg/dL).

*Statistically significant variables (p value < 0.05).

### Relationship between albumin-corrected calcium, metabolic syndrome, diabetes, and hypertension

Relationship between ACCA level and MetS, diabetes, and hypertension were analyzed by logistic regression in Table 3. ACCA level had positive association with MetS in the total population, male, and female, ORs were 1.20 (95% CI: 1.03–1.40 P = 0.022), 1.30 (95% CI: 1.03–1.64 P = 0.026), and 1.08 (95% CI: 0.87–1.34 P = 0.049) for model 2. ACCA level had positive association with diabetes in the total population, male, and female, ORs were 1.76 (95% CI: 1.41–2.20 P < 0.001), 1.68 (95% CI: 1.17–2.42 P = 0.005), and 1.86 (95% CI: 1.39–2.49 P < 0.001) for models 2. ACCA level had positive association with hypertension in the total population, OR was 1.15 (95% CI: 1.01–1.30 P = 0.037) for model 2.

**Table 3:** Relationship among ACCA, MetS, diabetes, and hypertension in the cross-sectional study.

	Metabolic syndrome				Diabetes mellitus				Hypertension			
	Model 1		Model 2		Model 1		Model 2		Model 1		Model 2	
	OR (95% CI)	P value	OR (95% CI)	P value	OR (95% CI)	P value	OR (95% CI)	P value	OR (95% CI)	P value	OR (95% CI)	P value
Total	0.99 (0.86–1.13)	0.835	*1.20 (1.03–1.40)	0.022	*1.49 (1.20–1.85)	<0.001	*1.76 (1.41–2.20)	<0.001	1.12 (0.99–1.28)	0.073	*1.15 (1.01–1.30)	0.037
Male	*0.76 (0.62–0.93)	0.006	*1.30 (1.03–1.64)	0.026	*1.43 (1.00–2.04)	0.050	*1.68 (1.17–2.42)	0.005	1.05 (0.87–1.27)	0.603	1.10 (0.90–1.33)	0.352
Female	1.12 (0.94–1.34)	0.217	1.08 (0.87–1.34)	0.490	*1.52 (1.15–2.00)	0.003	*1.86 (1.39–2.49)	<0.001	1.08 (0.91–1.29)	0.379	1.15 (0.96–1.37)	0.129

MetS, Metabolic syndrome; ACCA, Albumin corrected calcium; OR, Odds ratio.

Model 1: Adjusted for age, gender and body mass index (BMI); Model 2: Adjusted for age, gender, BMI, total cholesterol, triglycerides, LDL-C, creatinine, uric acid, serum albumin, proteinuria, and smoking status.

Response variables: MetS or diabetes or hypertension status; Explanatory variables: ACCA level (mg/dL).

*Statistically significant variables (p value < 0.05).

### Multivariate hazard ratios of future metabolic syndrome, diabetes, hypertension by serum calcium level

The results of using serum calcium level on prediction of future MetS, diabetes, and hypertension were shown in Table 4. High serum calcium level is associated with increased risk of incident MetS in the total population and male, hazard ratios (HRs) were 1.73 (95% CI: 1.07–2.79 P = 0.024) and 1.76 (95% CI: 1.07–2.89 P = 0.027) for model 2. Serum calcium level is associated with increased risk of incident diabetes in the total population and male, HRs were 2.51 (95% CI: 1.30–4.83 P = 0.006) and 2.86 (95% CI: 1.45–5.66 P = 0.003) for model 2. Serum calcium level is associated with increased risk of hypertension in the total population, male, and female, HRs were 1.76 (95% CI: 1.22–2.55 P = 0.003), 1.72 (95% CI: 1.17–2.50 P = 0.006), and 5.35 (95% CI: 1.14–25.03 P = 0.033) for model 2. There were too little samples for the development of MetS and diabetes in the female subgroup, the multivariate analysis was unable to perform.

**Table 4:** Multivariate hazard ratios of future MetS, diabetes, hypertension by serum calcium level.

	Metabolic syndrome				Diabetes mellitus				Hypertension			
	Model 1		Model 2		Model 1		Model 2		Model 1		Model 2	
	Events				Events				Events			
	HR (95% CI)	P value	HR (95% CI)	P value	HR (95% CI)	P value	HR (95%CI)	P value	HR (95%CI)	P value	HR (95% CI)	P value
Total	N = 109				N = 53				N = 165			
	*1.67 (1.08–2.58)	0.021	*1.73 (1.07–2.79)	0.024	*2.23 (1.21–4.12)	0.010	*2.51 (1.30–4.83)	0.006	*1.50 (1.06–2.13)	0.023	*1.76 (1.22–2.55)	0.003
Male	N = 86				N = 42				N = 129			
	*1.61 (1.03–2.50)	0.036	*1.76 (1.07–2.89)	0.027	*2.25 (1.21–4.19)	0.011	*2.86 (1.45–5.66)	0.003	1.35 (0.95–1.93)	0.096	*1.71 (1.17–2.50)	0.006
Female	N = 23				N = 11				N = 36			
	—	—	—	—	—	—	—	—	*12.42 (3.39–45.49)	<0.001	*5.35 (1.14–25.03)	0.033

MetS, Metabolic syndrome; HR, Hazard ratio.

Model 1: Adjusted for age, gender and body mass index (BMI); Model 2: Adjusted for age, gender, BMI, total cholesterol, triglycerides, LDL-C, creatinine, uric acid, serum albumin, proteinuria, and smoking status.

Response variables: Incident MetS or diabetes or hypertension; Explanatory variables: Serum calcium level (mg/dL).

*Statistically significant variables (p value < 0.05).

### Multivariate hazard ratios of future metabolic syndrome, diabetes, hypertension by albumin corrected calcium level

The results of using ACCA on prediction of future MetS, diabetes, and hypertension were shown in Table 5. High ACCA is associated with increased risk of incident MetS in the total population and the p-value was at borderline for statistical significance in the male population, HRs were 1.69 (95% CI: 1.04–2.74 P = 0.035) and 1.63 (95% CI: 0.99–2.69 P = 0.057) for model 2. ACCA is associated with increased risk of incident diabetes in the total population and male, HRs were 2.95 (95% CI: 1.43–6.07 P = 0.003) and 3.39 (95% CI: 1.57–7.33 P = 0.002) for model 2. ACCA is associated with increased risk of hypertension in the total population, male, and female, HRs were 2.07 (95% CI: 1.41–3.02 P < 0.001), 1.72 (95% CI: 1.17–2.50 P = 0.006), and 11.40 (95% CI: 3.22–40.44 P < 0.001) for model 2. There were too little samples for the development of diabetes in the female subgroup, the multivariate analysis was unable to perform.

**Table 5:** Multivariate hazard ratios of future MetS, diabetes, hypertension by ACCA level.

	Metabolic syndrome				Diabetes mellitus				Hypertension			
	Model 1		Model 2		Model 1		Model 2		Model 1		Model 2	
	Events				Events				Events			
	HR (95% CI)	P value	HR (95% CI)	P value	HR (95% CI)	P value	HR (95% CI)	P value	HR (95% CI)	P value	HR (95% CI)	P value
Total	N = 109				N = 53				N = 165			
	*1.774 (1.12–2.80)	0.014	*1.69 (1.04–2.74)	0.035	*3.01 (1.50–6.03)	0.002	*2.95 (1.43–6.07)	0.003	*1.93 (1.33–2.80)	0.001	*2.07 (1.41–3.02)	<0.001
Male	N = 86				N = 42				N = 129			
	*1.74 (1.08–2.80)	0.024	1.63 (0.99–2.69)	0.057	*3.46 (1.63–7.33)	0.001	*3.39 (1.57–7.33)	0.002	*1.61 (1.11–2.35)	0.013	*1.72 (1.17–2.53)	0.006
Female	N = 23				N = 11				N = 36			
	1.92 (0.24–15.50)	0.542	2.19 (0.22–21.66)	0.504	—	—	—	—	*9.46 (3.64–24.59)	<0.001	*11.40 (3.22–40.44)	<0.001

MetS, Metabolic syndrome; ACCA, Albumin corrected calcium; HR, Hazard ratio.

Model 1: Adjusted for age, gender and body mass index (BMI); Model 2: Adjusted for age, gender, BMI, total cholesterol, triglycerides, LDL-C, creatinine, uric acid, serum albumin, proteinuria, and smoking status.

Response variables: Incident MetS or diabetes or hypertension; Explanatory variables: ACCA (mg/dL).

*Statistically significant variables (p value < 0.05).

## Discussion

Both higher total serum calcium and higher ACCA were associated with a higher prevalence of hypertension. In addition, high serum calcium and ACCA were strong predictors of future hypertension. As far as we know, this is the first longitudinal study to use plasma calcium as a predictor of hypertension. There are cross-sectional studies that support our results. A study of 12,406 U.S. adults found a positive correlation between serum total calcium level and hypertension^10^. Another study that included 12,865 and 14,293 Norwegian men and women, respectively, also found a positive correlation between serum calcium and blood pressure^9^. The influx of calcium into the smooth muscle of the artery leads to muscle contracture which increases vascular resistance and therefore could lead to development of hypertension. In addition, plasma calcium may be associated with shorter telomere length, which is linked to increased coronary artery calcium. The calcium component of coronary plaques leads to negative remodeling, resulting in a narrower vessel lumen, causing major adverse cardiovascular events^14^. Moreover, a positive correlation has been found between serum calcium and cholesterol, which are two important risk factors for hypertension development^9^. Several previous studies have found a positive correlation between PTH concentration and blood pressure^15–17^. PTH induces the reorganization and production of collagen by aortic vessel smooth muscle cells, leading to vascular wall thickening^18^.

Previous studies have suggested no correlation or a negative correlation between serum calcium and hypertension. For example, the study conducted by Hazari *et al*. concluded that serum calcium had no effect on hypertension^19^. Unfortunately, their variables were not adjusted by age, BMI, total cholesterol, triglycerides, etc., as we have done.

Higher serum total calcium level and ACCA were associated with a higher prevalence of diabetes. High serum total calcium level and ACCA were good predictors of future diabetes in the total population and the male subgroup. There were too few events for development of diabetes in the female subgroup. Since the mean age of our study population was under average menopausal age, this result may be have been due to the protective effect of estrogen against insulin resistance^20,21^. The results are in accordance with most of the similar studies that have been conducted. Significantly higher serum calcium or ACCA level among diabetic subjects has been found in previous studies^11,12,22^. Sing *et al*. followed up 6096 participants for 59,130.9 persons-years while measuring serum total calcium and ACCA and found an association between elevated serum calcium concentration and incident diabetes^23^. Rooney *et al*. also obtained the same result by following 1516 participants for an average of 8.8 years^24^. There are three possible mechanisms to explain this result. First, serum calcium is associated with insulin resistance in adipocytes and skeletal muscle as a result of a decreased number of glucose transporters^24,25^. Calcium-dependent enzymes and calcium channels are responsible for glucose uptake after insulin stimulates muscle cells^12,26,27^. Second, hypophosphatemia and hypercalcaemia often present simultaneously^28^. Previous studies have concluded that the relationship between low serum phosphate level and reduced insulin sensitivity is a result of impaired phosphorylation of carbohydrate intermediates in the glucose metabolism pathway^29,30^. Finally, a negative correlation was discovered between serum calcium and beta-cell function^12^. Studies have shown that disturbance of calcium homeostasis leads to impaired insulin secretion of beta-cells^31^.

Positive associations were found between ACCA and prevalence of MetS in the total population and among each gender. Positive associations were found between ACCA and prevalence of MetS in both partially and fully adjusted model of the male population. The risk of MetS was positively correlated to high serum calcium level and ACCA in the total population. The risk of MetS was positively correlated to high serum calcium level in the male population. We have already discussed the correlation between blood pressure, insulin resistance and calcium level above. Here we will focus on three other components of MetS: waist circumference (WC), TG, and HDL-C. A cross-sectional study conducted by Saltevo *et al*. randomly included 4500 middle-aged men and women and found that serum calcium level was associated with all of the MetS components except HDL-C^13^. Ahlström *et al*. included 1016 healthy individuals and found a positive correlation between plasma calcium and WC. In addition, the more criteria of the NCEP for metabolic syndrome were met, the higher the parathyroid hormone (PTH) level and ACCA were. PTH was positively associated with BMI, WC, SBP, DBP, TG, and insulin resistance and negatively associated with HDL-C^17^. PTH plays a crucial role in the development of MetS components. A previous study revealed that individuals with higher PTH level had a higher chance of developing metabolic obesity^32^. Part of this can be explained by the insulin resistance effect caused by PTH, or serum calcium as we have mentioned above. Moreover, the dyslipidemia consequence of higher PTH level can also be attributed to central obesity^32^. A significant association has been found between serum calcium and unfavorable lipid profile, including triglycerides and HDL-C^33^. Conversely, serum calcium impairs cholesterol catabolism in the liver and increases lipid synthesis^34,35^. With regard to HDL-C, a previous study indicated no correlation between HDL-C and serum calcium^13^. To the best of our knowledge, the mechanism involved in the association between HDL-C and serum calcium is still not well understood. Some studies have hypothesized that the sterol regulatory element-binding protein (SREBP) pathway plays a key role in HDL-C^9,33,36^. In addition to the SREBP pathway, the relationship between PTH and HDL-C is again highlighted^17^. PTH level has been correlated with several inflammatory markers^37^. The link between inflammation and MetS has been reported, for example, an inflammatory diet pattern is associated with MetS and its components.

There are several limitations to our study. First, we did not measure ionized calcium, which is the most active form of calcium. Although we applied ACCA, there are studies that suggest ionized calcium is a better indicator of the serum calcium status^38,39^. Second, this would have been a more complete study if we had taken the level of PTH into account, since previous studies have shown a strong relationship between PTH level and MetS components^17,24^. Third, because serum calcium and phosphorous levels are closely related, we should also take the phosphorus level into consideration^28–30^. Fourth, there were too few events for the development of MetS and diabetes in the female subgroup. Fifth, we could not ensure all participants fasted for at least 8 hours. Last but not least, our study population was composed of mostly Chinese and lacked other ethnicities, such as Africans or Caucasians, who have shown different efficiencies on calcium metabolism^24^.

## Conclusion

Higher serum calcium and ACCA are associated with an increased risk of developing MetS, diabetes, and hypertension, especially among the male population. Serum calcium and ACCA are associated with increased prevalence of MetS, diabetes, and hypertension. Strength of the study includes 1. Large study sample was included 2. The adjustment for potential confounders were performed 3. The study included much discussion of the prior literature on the topic. Further research with international comparisons and discussion is needed to better establish the association between serum calcium level and cardiometabolic diseases.

## Methods

### Study design and population

We acquired data from participants who received health exams at Tri-Service General Hospital (TSGH), Taiwan during the period 2010 to 2016. There were 27,364 eligible subjects aged 20 years of age and older. Our study was approved by Institutional Review Board (IRB) of TSGH. The IRB waived the need to obtain individual informed consent because the data were analyzed anonymously. Participants enrolled were analyzed according to following steps. First of all, baseline characteristics were determined. In the cross-sectional study, optimal cut-off values for serum calcium and ACCA to predict the future development of cardiometabolic diseases were obtained respectively for gender difference using receiver operating characteristic curves (ROC). Next, the exclusion criteria of the cohort study included those with baseline MetS, diabetes, hypertension or other systemic diseases (n = 5,234), subjects with only one visit (n = 10,230), subjects with insufficient data at end-point (n = 1,820). Finally, there were 10,080 participants. Multivariate longitudinal analysis were conducted to examine whether higher serum calcium or ACCA level were associated with higher incident rate of cardiometabolic diseases.

### Defining criteria for metabolic syndrome

According to MetS criteria defined by the 2005 revised NCEP ATP III, MetS is comprised of at least 3 of the following 5 components: (1) plasma triglycerides ≥150 mg/dl (1.7 mmol/L) or under treatment (2) HDL cholesterol <40 mg/dl (1.03 mmol/l) in men, <50 mg/dL (1.29 mmol/l) in women or under treatment (3) systolic blood pressure ≥130 mm Hg and/or diastolic blood pressure ≥85 mm Hg or under treatment (4) fasting plasma glucose ≥100 mg/dl, under treatment, or previously diagnosed with diabetes mellitus (5) waist circumference cutoffs modified for Asian populations, ≥ 90 cm in men or ≥ 80 cm in women^40^.

### Definition of hypertension

Based on the Seventh Report of the Joint National Committee on Prevention, Detection, Evaluation, and Treatment of High Blood Pressure, hypertension is defined as blood pressure higher than 140/90 mmHg or subjects have already been taking antihypertensive agents^41^.

### Definition of type 2 diabetes

Based on the 2019 American Diabetes Association (ADA) “Standards of Medical Care in Diabetes”, type 2 diabetes is defined as random plasma glucose ≥200 mg/dL with symptoms (polydipsia, polyphagia, polyuria, and weight loss) or raised value measured twice. Fasting plasma glucose ≥126 mg/dL can also lead to diagnosis. In addition, those already taking anti-diabetic agents are also considered to have type 2 diabetes^42^.

### Assessment of covariates

Health examination includes physical and mental status evaluation, anthropometric measurements, and comprehensive biochemistry tests. Participants were fasted for at least 8 hours prior to phlebotomy. Biochemistry tests are composed of blood cell counts, concentration of blood urea nitrogen, serum creatinine, aspartate transaminase (AST), alanine transaminase (ALT), total calcium, serum albumin, fasting glucose, total cholesterol, high-density lipoprotein cholesterol (HDL-C), low density lipoprotein cholesterol (LDL-C), triglycerides, serum uric acid, and urinalysis. Fasting blood sample was drawn between 7:30 am and 11:00 am and stored in a 4 °C refrigerator immediately^43^. We applied the o-cresolphthalein complexone method to obtain serum calcium level. ACCA was calculated by using [serum calcium + 0.8 × (4–serum albumin)]. The unit for serum calcium and ACCA is mg/dL. Blood pressure was taken after participants have received at least 10-min of rest. Participants were seated with a sphygmomanometer placed on right arm. Waist circumference was measured at the middle point between iliac crest and 12^th^ rib.

### Statistical analysis

SPSS (version 18.0 for Windows; SPSS Inc., Chicago, IL, USA) was used as the software for statistical analysis. Optimal cut-off values of serum calcium level and ACCA for the male and female participants respectively were calculated by ROC. At baseline, participants were divided into two subgroups according to the cut-off values of serum calcium and ACCA. Student’s t-test was performed to evaluate the difference of continuous characteristics at baseline between the two groups. Data was shown as mean ± standard deviation and the distribution normalized with Kolmogorov-Smirnov test. Two-sided P-value less than 0.05 was considered statistically significant. Logistic regression for calculating OR was used to determine association between serum calcium or ACCA level and MetS, diabetes, hypertension at baseline. Univariate and multivariate Cox proportional hazard models were applied to obtain the HR of both groups during the follow-up period to find out whether serum calcium or ACCA exceeding the cut-off values were associated with increased risk of developing MetS, diabetes, and hypertension. In our study, two models were investigated as follows. Model 1 was adjusted for age, gender and BMI. Model 2 was adjusted for age, gender, BMI, total cholesterol, triglycerides, LDL-C, creatinine, uric acid, serum albumin, proteinuria, and smoking status.
